# An Easily Effectual Method of Artificial Respiration

**Published:** 1875-09

**Authors:** J. D. Mattison

**Affiliations:** Chester, N. J.


### AN EASILY EFFECTUAL METHOD OF ARTIFICIAL RESPIRATION.

BY J. D. MATTISON, CHESTER, N. J.

Among the many exigencies arising in the accouchment chamber, more, oftimes, are fraught with more importance than those pertaining to the thorough establishment of respiration in the newly born babe.

Momentous interests, affecting the worldly weal of many, may hang on the performance of this function, be the time of its complete carrying on never so limited, and, as well known among the adherents of a certain ecclesiastical belief, the establishment thereof sufficiently long to permit a peculiar ritual performance, entitles the subject to a sharing in those joys that are to be. Aside from these considerations, the advent of the little stranger awakens emotions in the maternal bosom, such as only a parent can know, and to have fond hopes and loving anticipations dashed ruthlessly down may give rise, in highly impressionable temperaments and under unusual circumstances, to symptomatic manifestations seriously imperiling a life even more valuable.

It behooves him, therefore, who takes upon himself the duties of the lying-in-apartment to be fully alive to all its demands in this direction, and to meet, promptly, with the most efficient means at his disposal, whatever of an emergency may arise.

Every qualified practitioner is supposed to be cognizant of the various conditions standing in a causative relation to this state of apparent death, and their variety necessitates a varied plan of operative procedures, looking to resuscitation. Often simple measures suffice, but again it is only after the most assiduous attention that we are rewarded by the first feeble evidences of an independent existence.

Among the different means of restoring asphyxiated infants, artificial respiration, after the manner of Hall or Sylvester, has long held a foremost place and been successfully resorted to after the failure of other measures. More recently, another plan, that of Schultze, has come into notice, and as it may be unfamiliar to many practitioners, and is, undoubtedly, of value, the following description will perhaps be acceptable:

"The operator seizes the child under the arms, the index finger of each hand in the armpit, the thumbs over the anterior portion of the trunk, the remaining fingers placed along the back, which is turned toward the operator, while the head is steadied between the palms of the hands. As the operator stands, the child so held, is allowed to swing between the outspread knees. The tractions thus made in both directions upon the ribs by the pectoral muscles above, and the abdominal muscles below, produce the widest separation of the ribs, while the weight of the liver causes descent of the diaphragm and thus inspiration is produced. Next, with extended arms, the operator swings the child upward until the breech and legs fall forward toward the abdomen. When the body is thus doubled up, the ribs close together, the diaphragm is pushed upward and forcible expiration takes place, driving out through the mouth and nostrils great quantities of mucus and fluids—when respiratory movements have taken place prior to birth—from the air passages. Still keeping the arms extended, the child should be allowed after a few moments (?) to swing back between the legs. In this way expiration and inspiration are to be maintained until spontaneous respiration occurs. As the temperature is apt to fall during the swinging movements, warm water should be kept handy in which to occasionally plunge the child."

We desire to call attention to another method, the value of which we have, on more than one occasion, domonstrated to our intense satisfaction, and which has, we think, advantages over that just detailed, in being more simple, seemingly not so rough, and, we judge, equally efficacious.

We disclaim originality; credit in that direction is due to Prof. Harvey L. Byrd, of Baltimore, who, in an article entitled "a speedy

Method in Asphyxia," published in the Baltimore Med. Journal, Nov., 1870, and reprinted in the Half Yearly Compendium of Medical Science, Jan., 1871, described the modus operandi, and cited cases confirming its value.

The method we present has, however, some modifications of Dr. Byrd's, and is as follows :

"The infant upon its back firmly grasp the outer thigh, the index finger and thumb encircling, and the inner limb resting on the forearm, while the little finger is extended as far as possible up the back to form a fulcrum with the corresponding finger of the opposite hand. In the hollow formed by the thumb and fore-finger of the right hand allow the neck of the infant to rest with the palm under the shoulders and the little finger extending down the back to meet its fellow of the other hand. Now, gently and regularly depress the vertex and inferior extremities as much as practicable below the horizontal, say forty-five degrees, thus facilitating inspiration, and after a proper interval, elevate them to the same extent forming a concavity of the chest and thereby favoring expiration, continue these movements without interruption, taking care to permit no impediment to the exit and entrance of air during the upward and downward movements of the head and chest, and also exercising caution against too much lateral motion of the head during their continuance. The conjoined use of Desormeaux's douche, or a little cold water dashed occasionally on the epigastrium, will tend to enhance the efficacy of this method ; indeed, its employment not at all precludes the use of whatever auxiliary measures may be deemed advisable.

In Prof. Byrd's plan, the uluar edges of the hands are placed in contact to form the fulcrum beneath the infant's back, the thumbs extended, and the radial borders being alternately elevated and depressed simulate the normal respiratory act. Modified, as above, its advantages are: a much firmer hold of the body is obtained, thereby diminishing the chances of slipping; it is less fatiguing to the operator, and it obviates a necessity for an attendant to prevent any considerable departure of the head from its antero-posterior axis with the vertebral column.

We speak whereof we know, regarding the value of this method, having had ample evidence in that direction. Among other cases, notes of a remarkable instance of infantine asphyxia in which it was, we confidently believe, the life-saving instrumentality, not only in establishing respiration after a notably protracted absence, but in maintaining existence through a perilously prolonged narcosis, go far in substantiating the statement of its originator that "it is *par excellence* the remedy in the asphyxia of newly born infants."

We have met those of large experience who were unacquainted with it. In the hope that it may be given a wide publicity, and its merits made available, we re-present and commend it to the profession.

